# Management of Cervically Fractured Central Incisors: A Multidisciplinary Approach

**DOI:** 10.2174/1874210601812010238

**Published:** 2018-03-30

**Authors:** Rakesh. R. Rajan, Sapna Chandira Muddappa, Priya Rajendran, Ashok Sukumaran Nair

**Affiliations:** 1 Amrita School of Dentistry, Amrita University, Aims Campus, Kochi - Conservative Dentistry & Endodontics, Ernakulam, Kerala, India; 2 Department of Conservative Dentistry & Endodontics, Malabar Dental College, Malapuram, Kerala, India

**Keywords:** Tooth fracture, Gingival margin, Crown restoration, Orthodontic extrusion, Alveolar ridge, Maxillary anterior teeth

## Abstract

**Introduction:**

Fracture of tooth structure at or below the gingival margin compromises rehabilitation and hampers esthetics and function.

**Management:**

Management of such cases by a post-core and crown restoration, or periodontal surgery or orthodontic extrusion alone may not always suffice in attaining a good result.

**Case Report:**

A multi-disciplinary approach which includes all of the above mentioned procedures helps in long term success.

**Conclusion:**

Careful case evaluation, treatment planning and meticulous attention to detail are the keys to the best treatment outcome.

## 1 INTRODUCTION

Fracture of a tooth at a level apical to the gingival attachment presents a huge challenge for restorative management. In such situations, often the tooth is considered to have poor esthetic and functional prognosis and is indicated for extraction. Periodontal crown lengthening has been suggested as a treatment option but it usually compromises esthetics [1]. Orthodontic extrusion/ forced eruption came into the foray as an alternative or adjunct to periodontal crown lengthening [2]. The factors that dictate treatment planning in such cases are:

- Apical closure (status of root closure)
- Eruption status
- Comminuted fracture
- Sub gingival fracture (level of fracture)
- Above the alveolar ridge
- Below the alveolar ridge

This article outlines the successful management of a tooth with subgingivally extending crown fracture, by a combination of periodontal and orthodontic interventions, reflecting a true multidisciplinary approach.

## 2 CASE REPORT

A thirteen year old male patient reported to the dental office, with the complaint of dislodged prosthetic crowns in upper front teeth region. Anamnesis revealed a history of root canal treatment and full crown restorations in the maxillary anterior teeth.

**Fig. (1):**
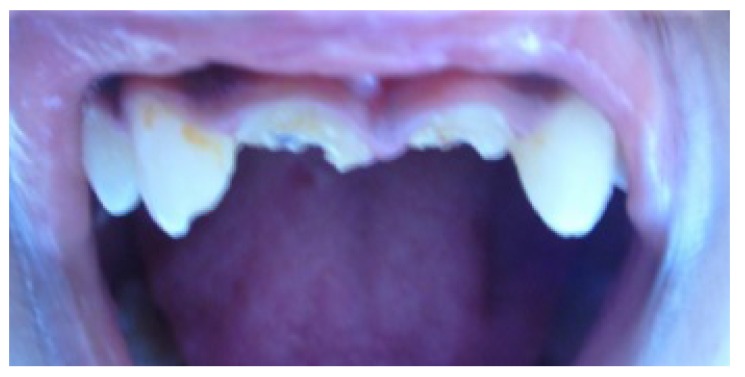
Pre- operative photograph.

Clinical examination revealed dislodged metal ceramic crowns on 11 and 21, with the fractured tooth structure lodged within the crowns (Fig. **1**). The average probing pocket depth was 2.5 mm and the width of attached gingiva was 3 mm.

**Fig. (2):**
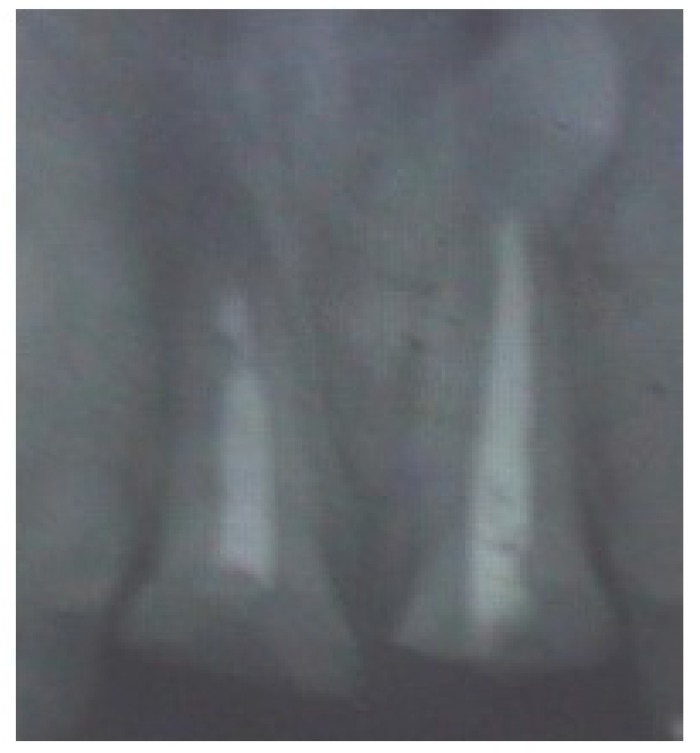
Pre-operative radiograph.

Radiographic examination disclosed a suboptimal obturation of the root canals of both 11 and 21 (Fig. **2**). Fracture of the teeth was seen to be at the level of the alveolar bone crest. Root length was assessed to be adequate to achieve 1:1 crown-root ratio following orthodontic extrusion.

## 3 MANAGEMENT

**Fig. (3):**
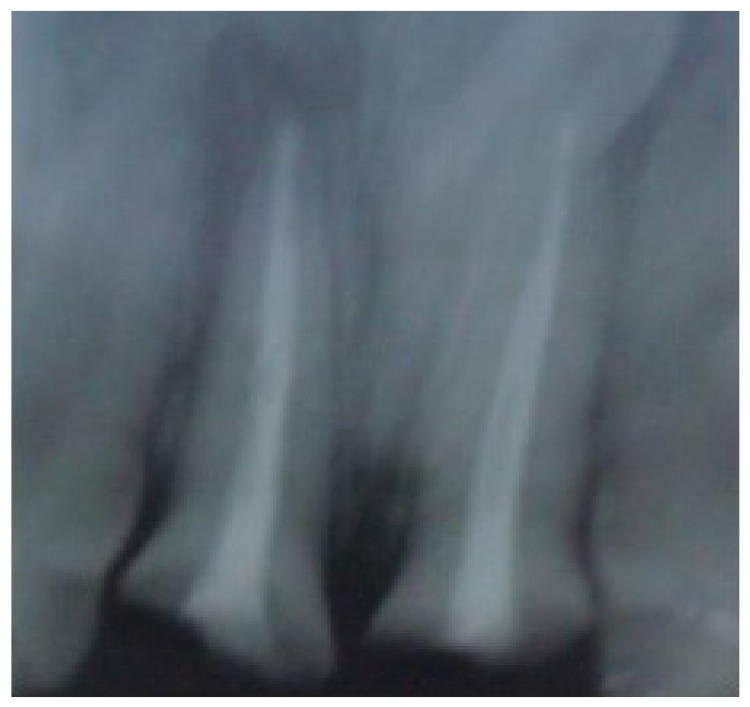
Retreatment done on 11,21,

**Fig. (4):**
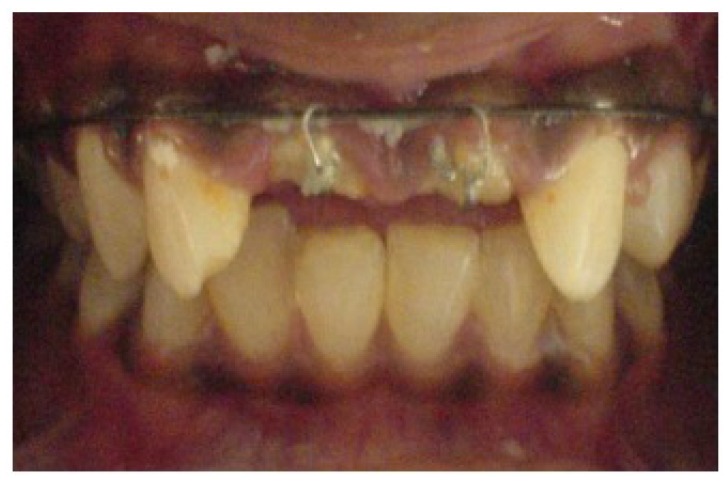
“J” hook luted in 11,21.

**Fig. (5):**
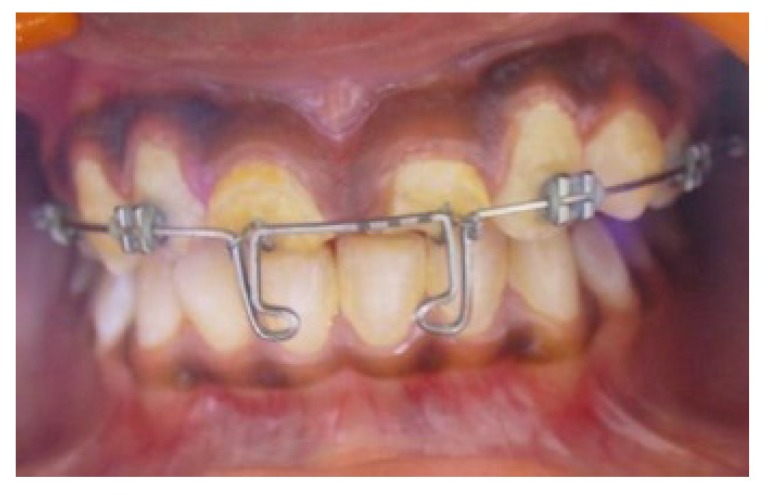
Orthodontic extrusion completed.

Non-surgical endodontic retreatment was done in 11 and 21, and both teeth were obturated by lateral condensation (Fig. **3**). Post spaces were then prepared to a depth of 11mm and size 3 peeso reamer (Mani Inc Japan). J hooks fabricated in 19 gauge stainless steel wire (Sendent Inc, TN,India) were luted within the prepared post spaces of both teeth. The patient was referred to the Department of Orthodontics for extrusion and labial movement of 11 and 21. 0.022 Slot with MBT prescription (3M Unitek, Monrovia, USA) were placed in the upper arch. (Fig. **4**) After initial alignment & leveling, an “L” hook was placed with 19 x 25 (SS) incisally and an active ligature tie was given to the “J” hook, which was then activated at 3 week intervals (Fig. **5**). Orthodontic appliance was in place for 14weeks, by when the adequate height had been achieved, and the orthodontic appliance was debonded.

**Fig. (6):**
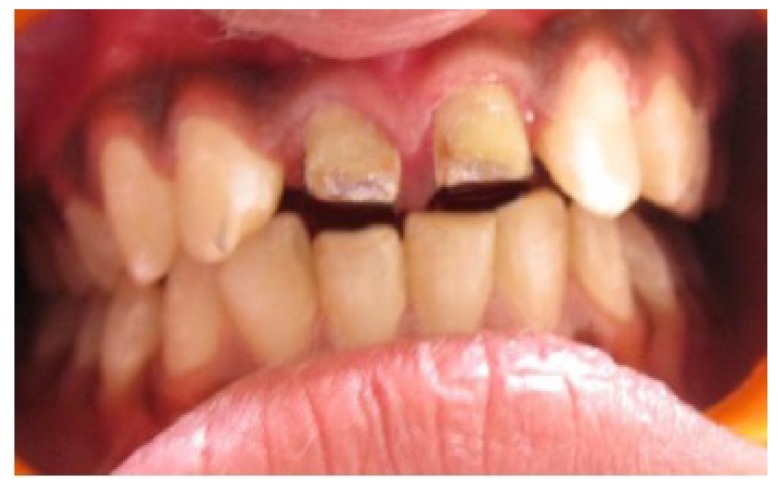
Crown lengthening achieved.

At this stage the probing pocket depth was 3 mm and the width of attached gingiva was 4 mm. Crown lengthening with osteotomy and gingivectomy was planned. This would expose an additional 1 mm of tooth structure for obtaining a ferrule and also help esthetic correction of the gingival levels. The attachment of the labial frenum was noted to be high on the ridge, which was also planned for simultaneous correction in conjunction with the crown lengthening (Fig. **6**).

**Fig. (7):**
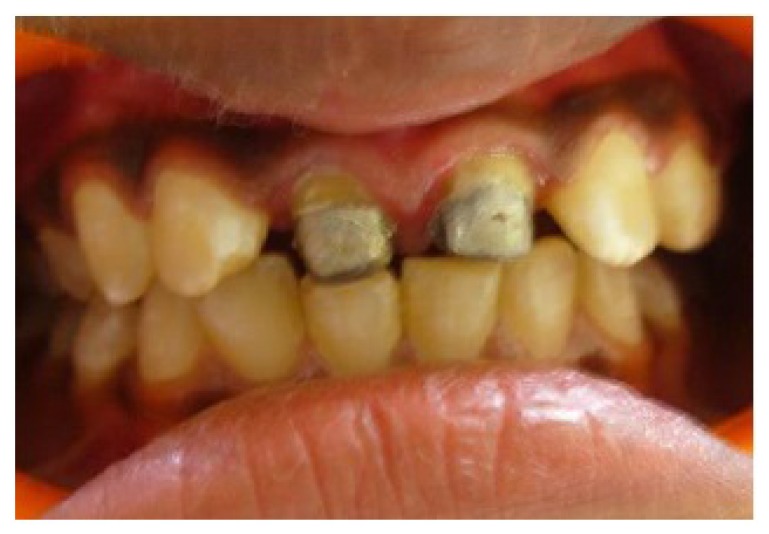
Cast post-core luted on 11,21.

**Fig. (8):**
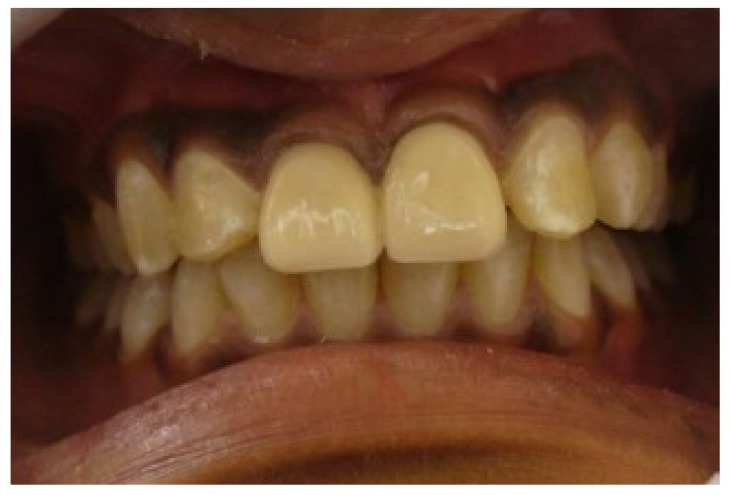
Porcelain fused to metal crown luted on 11,21.

**Fig. (9):**
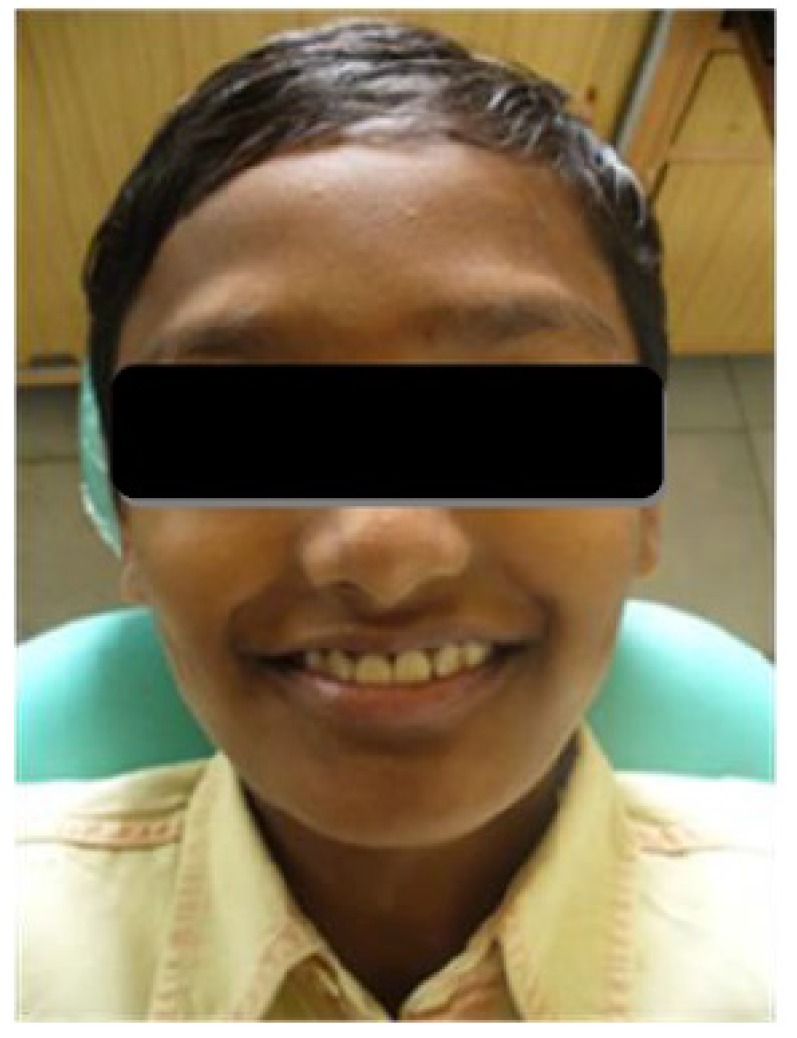
Post operative view.

**Fig. (10):**
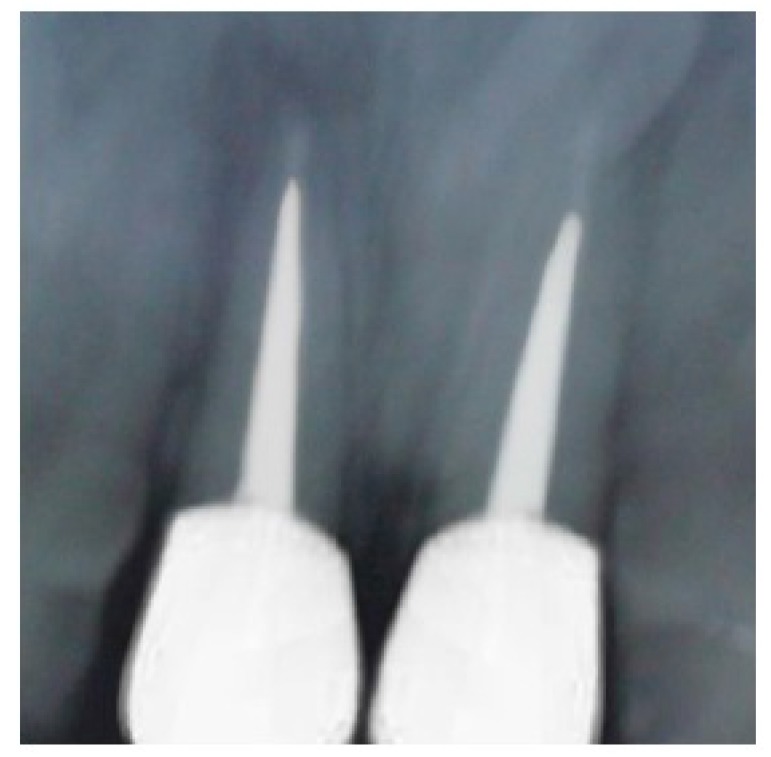
Six month follow-up radiograph.

 Following healing, cast posts were fabricated on 11 & 21 (Fig. **7**). Heat cure acrylic temporary crowns were given after tooth preparation till the passive eruption was complete. After 6 months metal ceramic crowns were luted on 11 & 21 (Figs. **8**-**10**).

After initial alignment & leveling, an “L” hook was placed with 19 x 25 (SS) incisally and an active ligature tie was given to the “J” hook, which was then activated at 3 week intervals (Fig. **5**). Orthodontic appliance was in place for 14 weeks, by when the adequate height had been achieved, and the orthodontic appliance was de-bonded.

At this stage, the probing pocket depth was 3 mm and the width of attached gingiva was 4 mm. Crown lengthening with osteotomy and gingivectomy was planned. This would expose an additional 1 mm of tooth structure for obtaining a ferrule and also help esthetic correction of the gingival levels. The attachment of the labial frenum was noted to be high on the ridge, which was also planned for simultaneous correction in conjunction with the crown lengthening.

Following healing, cast posts were fabricated on 11 & 21 (Fig. **6**). Heat cure acrylic temporary crowns were given after tooth preparation till the passive eruption was complete. After 6 months metal ceramic crowns were luted on 11 & 21 (Fig. **7**).

## 4 DISCUSSION

Dental trauma, and ensuing fracture of the permanent anterior teeth, can be one of the most stressful situations in a dental practice, demanding a comprehensive treatment planning. Complications persist with uncertainties in prognosis and treatment time, while long-term management can be complex [3].

The first step for control of inflammation and pain management is a well-executed root canal treatment. This would also be the foundation upon which any reconstructive treatment on the tooth can be based [4]. In this reported case, a retreatment had to be planned because of the unsatisfactory obturation, evident radiographically. Assessment following root canal retreatment showed the inadequate height of the remaining tooth structure to support a restoration. Hence, the options of surgical extrusion, forced orthodontic extrusion and periodontal surgery for crown lengthening were considered. Simple surgical crown lengthening would involve additional resection of the bone of the teeth adjacent to the tooth that is to be lengthened, making it an invasive procedure at large. However, such osteotomy could be avoided by the use of orthodontic extrusion [5]. Orthodontic extrusion is a biological way to expose sound tooth structure, but it often requires prolonged treatment. Rapid coronal movement with intense force provides faster clinical results, but has limited indications [6]. Rapid orthodontic therapy is known to alter the position of the tooth but the bony architecture does not follow the extruded tooth [2]. When stronger traction forces are exerted, as in rapid extrusion, coronal migration of the tissues supporting the tooth is less pronounced because the rapid movement exceeds their capacity for physiologic adaptation [5]. Hence, bone and gingival levels are required to be corrected surgically after orthodontic extrusion, this being one of the few disadvantages of the procedure. Korayem et al recommended forces of 15 and 50 cN for the anterior and posterior teeth, respectively, and a slow and steady extrusion rate of ≤ 2.0 mm/month [7]. After a latency period of a few days to a few weeks, including a period of hyalinization, slow extrusion occurs at a rate of approximately 1 mm or less per week [5]. This protocol involving a relatively longer duration and lighter forces was applied in the present case.

Stable anchorage is a pre-requisite for orthodontic movement with fixed appliances [8]. Dental implants, mini-plates and mini-screws are excellent orthodontic skeletal anchorage devices for orthodontic tooth movements including forced eruption [9]. In the present case, a more cost-effective and simpler alternative was applied.

Several surgical techniques have been suggested to achieve an accelerated extrusion, enabling the treatment to be completed within shorter durations. Wilcko’s technique combining fixed orthodontic appliances, labial and palatal/lingual corticotomies, and bone grafting, was reported to have a rapid tooth movement at a rate of 3-4 times greater than the conventional technique. However, the invasiveness of the procedure requiring full mucoperiosteal flaps constitutes a serious drawback [10]. Less invasive procedures like Corticision [11], Piezocision [12] and Piezopuncture [13] have been reported in the literature, but long-term results are as yet unavailable [10].

The other important consideration is the biologic width. It is defined as the physiologic dimension of the junctional epithelium and connective tissue attachment and is approximately 2 mm [14]. Infringement of the biologic width by placement of a restoration within this zone may result in gingival inflammation, pocket formation and alveolar bone loss [15].

In the present case, following extrusion, a 3-fold periodontal surgery was planned for several purposes. It included a minimal osteotomy and gingivectomy to provide a 2mm circumferential ferrule, as well as esthetic correction of the gingival levels in the anterior segment. Circumferential fibrotomy was done to sever the periodontal ligament attachments to aid in retaining the newly attained position of the tooth [16]. Frenectomy was also done to correct the high frenal attachment. This would provide for the best esthetic and functional result in the end.

The use of metal posts is justified by various studies showing their superior fracture resistance [17]. Since the amount of tooth structure remaining was minimal, a custom-made cast post and core was adjudged to be suitable for the case . The permanent restoration was delayed until it was ensured that the dentogingival junction is located entirely on cementum by passive eruption, to avoid esthetic discrepancy at a later time [18].

## CONCLUSION

A multidisciplinary approach can help save teeth which would otherwise be indicated for extraction. The key to success are good treatment planning and the dedication of the dentist to reassure and motivate the patient throughout the course of treatment. It is also mandatory to institute a strict and regular recall regimen to guarantee the long term prognosis.
